# Prevalence of Breast Self‐Examination and Associated Factors Among Female Nursing and Midwifery Trainees in Gushegu Municipality, Ghana

**DOI:** 10.1002/hsr2.72080

**Published:** 2026-05-13

**Authors:** Karima Mohammed, Gilbert Abotisem Abiiro, Robert Kokou Dowou, Frank Dadzie, Gifty Apiung Aninanya

**Affiliations:** ^1^ Nursing and Midwifery Training College Gushegu Northern Region Ghana; ^2^ Department of Health Services Policy, Planning, Management and Economics School of Public Health University for Development Studies Tamale Northern Region Ghana; ^3^ Department of Epidemiology and Biostatistics Fred N. Binka School of Public Health, University of Health and Allied Sciences Hohoe Volta Region Ghana; ^4^ Department of Social and Behavioural Change School of Public Health University for Development Studies Tamale Northern Region Ghana; ^5^ Department of Global & International Health School of Public Health University for Development Studies Tamale Northern Region Ghana

**Keywords:** attitudes, breast cancer, Gushegu Municipality, Ghana, knowledge, nurses, practice, self‐examination

## Abstract

**Background:**

Breast cancer poses a significant global public health challenge. While breast self‐examination (BSE) is a valuable tool for early detection, a notable gap exists in the literature regarding its practice among nursing and midwifery students. This study sought to identify the factors predicting BSE knowledge and practice among female students at the Gushegu Nursing and Midwifery Training College in Ghana.

**Methods:**

A quantitative cross‐sectional study was conducted with 135 nursing students. A structured questionnaire was used to collect data, and a multistage sampling technique was employed. The collected data were analyzed using SPSS 25.0, with descriptive and inferential statistics used to determine associations. A *p*‐value of less than 0.05 was considered statistically significant.

**Results:**

The study found that most participants were aware of breast cancer (94.9%), with media serving as the primary source of information (38%). A high percentage of students (95.6%) were knowledgeable about BSE, and 65.2% demonstrated good procedural knowledge. Notably, 71.5% of the respondents reported practicing BSE. The strongest predictors of BSE practice were: being a registered midwife trainee (AOR = 0.08, 95% CI: 0.015–0.443), engaging in physical exercise (AOR = 16.7, 95% CI: 2.43–114.9), and having a family history of breast cancer (AOR = 6.54, 95% CI: 0.42–101.1). Other significant predictors included awareness of breast cancer, awareness of BSE, perceived importance, and learning about BSE from professional sources.

**Conclusion:**

The findings suggest that most nursing and midwifery students in this study possess good knowledge of and actively practice BSE. However, the results underscore the need for targeted educational interventions to reinforce proper BSE frequency and technique to further improve breast cancer early detection efforts.

## 1 Introduction

Breast cancer is an increasing global health concern, particularly among women aged 15–39 years, accounting for 5.6% of all invasive cancers and being a leading cause of cancer‐related deaths in many countries [1]. In 2020, there were approximately 2.3 million diagnoses and 685,000 related fatalities of breast cancer worldwide [1]. The disease disproportionately affects low‐ and middle‐income nations, where 324,000 victims reside, and it is the most frequent disease among women in Sub‐Saharan Africa (SSA) [1]. In 2020, out of the 19.3 million new cases of cancer worldwide, 16.8% occurred in SSA. Breast cancer also accounted for 15% of the 9.9 million cancer‐related deaths worldwide, with SSA accounting for 12.1% of those cases [1]. Specific examples highlight its impact: in 2013, over 11,000 women in the United States were diagnosed with invasive breast cancer, leading to about 1000 deaths [1, 2], while Brazil recorded 19,105 breast cancer deaths among young women between 1996 and 2017 [3]. From 1996 to 2017, the incidence of breast cancer in young women aged 20–29 years increased by 2.2% per year [4, 5].

In Ghana, breast cancer is a significant public health issue and the leading cause of cancer‐related deaths among women. Annually, approximately 2260 new cases of breast cancer are diagnosed, resulting in an estimated 1021 fatalities, and the incidence rate ranges from 15.2 to 35 per 100,000 women [6]. Regional data underscores this problem as health facilities in Ho, Volta Region, saw cases rising from 144 in 2013 to 252 in 2015, while in the Northern Region, 10 out of 900 people screened in 2020 were diagnosed with breast cancer [6, 7, 8, 9]. A major concern is that many women seek medical care for breast cancer too late, when effective treatment is no longer possible.

The International Breast Cancer Month, observed in October, aims to boost awareness and promote early diagnosis and treatment methods. Mammography, clinical breast examination (CBE), and breast self‐examination (BSE) are key screening techniques. BSE is highlighted as the most accessible, affordable, and straightforward method, making it ideal for resource‐limited settings like Ghana, where it can frequently facilitate early breast cancer diagnosis, with over 90% of cases potentially detectable by women themselves. The American Cancer Society recommends monthly BSE for women aged 20 and older [8, 10].

Despite its benefits, the nursing and medical professions often inadequately address BSE as they tend to focus more on older or vulnerable women, and frequently overlook younger demographics [9]. This oversight, coupled with a general lack of knowledge and appropriate attitudes toward breast cancer among women in developing countries like Ghana, contributes to low detection rates, delayed diagnoses, and the presentation of advanced breast cancer cases [11]. Although nursing students in Ghana receive basic health education on breast cancer and early detection, primarily through lectures [12]. There remains a critical need to evaluate their specific knowledge levels regarding breast cancer and their practices related to BSE.

While existing studies have explored BSE and related factors, they generally do not specifically target nursing trainees. A study on female health personnel at Kathmandu Medical College and Teaching Hospital, Nepal, found that while most had a positive attitude toward BSE, their knowledge of the practice was generally average or poor [13]. Another study conducted in Ethiopia showed that the practice of BSE among female health professionals was low [14]. Also, a meta‐analysis of 12 studies involving 4129 female healthcare workers in Ethiopia revealed that the pooled prevalence of BSE was 56.31% and higher BSE practice was observed among certain healthcare groups (58.60%). Factors significantly associated with BSE practice included good knowledge, positive attitude, and a family history of breast cancer [15]. In Ghana, a cross‐sectional study done in the Volta Region among 385 women aged 15–49 years found that while 88.3% were aware of breast cancer, only 64.9% had adequate knowledge of it, and 37.6% practiced BSE, with over half lacking correct BSE skills. There was also significant link between breast cancer knowledge and BSE practice, and also between age and BSE practice, with older women practicing BSE less frequently [16]. While most female students in the Sunyani Municipality are aware of BSE, significant gaps in technical knowledge and practice persist, with age being a key predictor of awareness [17]. Also, a study conducted in the Volta Region found that while most women were aware of breast cancer, only 37.6% practiced BSE, with knowledge significantly linked to practice; additionally, older women were less likely to perform BSE [18]. Also, a scoping review highlights significant discrepancies in breast cancer screening uptake across different regions in Ghana, and despite the well‐known benefits of early detection, the utilization of screening methods remains very low throughout the country, primarily due to various barriers that differ across regions [19]. These considerable differences across populations underscore the necessity for further investigation into the underlying reasons for such disparities in BSE prevalence.

Research on determinants of BSE in Northern Ghana has mainly focused on females with diverse backgrounds. A study examined knowledge of breast cancer risk factors and BSE practices among 1122 women in Tamale, Northern Ghana, from various occupational backgrounds [20]. This study suggested that most participants were knowledgeable about breast cancer (93.1%) and BSE (87.6%), and although 76.4% had been taught how to perform BSE, only 37.2% practiced it regularly [20]. Research on BSE among nursing students in Ghana's Northern Region is limited. Existing studies indicate high awareness (89%) and positive attitudes (94.4%) toward BSE, with most students recognizing its importance in early cancer detection and having prior education on the topic (91.3%) [12]. While a study in Chereponi established that BSE knowledge significantly increases the likelihood of practice, actual usage rates among women remain low. In the Chereponi area, BSE practice is frequently hindered by psychological barriers like embarrassment and discomfort, alongside deep‐seated cultural norms [21]. Additionally, lack of accessible mammography services further complicates proactive breast health management [20, 22]. Previous research has included women in general, secondary and tertiary students, as well as female university students [23, 24, 25], but few studies have specifically examined nursing trainees, particularly in rural settings. Some evidence suggests that nursing students possess better knowledge and clinical experience related to BSE, with notable differences observed between nursing and teaching trainees in terms of BSE knowledge and practice. However, these studies mainly focused on urban populations and employed varied methodologies [22, 23, 24]. This study addresses critical research gaps by examining the knowledge and practices regarding BSE among nursing and midwifery trainees in the rural Gushegu district. Rural communities often face distinct barriers to early cancer detection, including cultural beliefs and limited access to healthcare facilities, which differ significantly from urban settings. Trainees in rural settings are future healthcare providers who can influence community practices. Assessing their knowledge and practices on BSE can help develop targeted interventions. The current study, therefore, aims to address gaps by assessing the prevalence of BSE and its associated factors among nursing students in Gushegu, a rural area in Northern Ghana.

## 2 Methodology

### 2.1 Study Design and Setting

This study employed an analytical cross‐sectional design with a quantitative approach. This study allowed for the investigation of both exposure and outcome simultaneously and was chosen for its cost‐effectiveness. The Gushegu Municipality is one of the Northern Region's 16 Metropolitan, Municipal and District Assemblies [26]. The municipality is home to numerous educational institutions, including 24 kindergartens/nurseries, 115 primary schools, 31 junior high schools, and one senior high school. The municipality has one hospital located in Gushegu, the municipal capital, which serves as a referral hospital for the area [27]. There are also two health centers, one Reproductive and Child Health Clinic in Gushegu, and nine Community Health Planning System (CHPS) compounds. Additionally, the municipality boasts a Health Training School in Gushegu that offers a Diploma in Midwifery and Registered Nurse Assistant Clinical programs. The college was established in October 2012 and currently has 22 tutors and a student population of 407 [28].

### 2.2 Study Population

The study population consisted of female trainee nurses, aged range from 18 to 33 years, drawn from the Gushegu Nursing and Midwifery Training College. The students' population is about 407 [29].

Participants included in this study were females at the Gushegu Nursing and Midwifery College in the Northern Region, who were between the ages of 18 and 33 years and voluntarily consented to participate in the study.

### 2.3 Sample Size

Using the prevalence of BSE of 43.1% from a study conducted in the Tamale Metropolis [30], the sample size for this study was determined using the Cochran formula, *n* = $\frac{z^{2}(\mathrm{pq})}{d^{2}}$. This calculation yielded a sample size of approximately 376. However, due to logistical considerations and participant availability, a total of 135 students ultimately participated in the study.

### 2.4 Sampling Techniques

The study employed a multistage sampling technique to select participants. The first stage involved selecting one Nursing and Midwifery Training College from the four colleges in the Northern Region using simple random sampling to ensure unbiased selection.

In the second stage, within the selected college, all relevant programs were considered, with each program comprising three levels: first, second, and third year. Each level was further divided into two classes, labeled “A” and “B.” Based on estimations, each class had at least 50 female students, out of a total of approximately 120 students per class.

In the third stage, eight classes out of the 12 classes across all levels and programs in the selected college were randomly chosen. This was done using simple random sampling, with each class representing a cluster.

### 2.5 Data Collection Tool and Procedures

A structured questionnaire was used to collect the data. This was developed after reviewing existing questionnaires/data collection tools [20] that have been used in previous studies [31, 32]. The instrument was divided into three sections: Section A dealt with the demographic and socioeconomic features of the students, Section B dealt with knowledge of breast cancer and BSE, and Section C dealt with practices of self‐breast examination. To ensure the reliability and validity of the data collected, pre‐testing of the data collection instruments was conducted among 30 nursing and midwifery trainee students from the Nalerigu Nursing and Midwifery Training College. This was important because the pilot study participants shared similar characteristics with those at Gushegu Nursing and Midwifery Training School. The pre‐testing helped in refining and restructuring the data collection tool to enhance its consistency and effectiveness in eliciting the appropriate information.

The instrument was later self‐administered under the supervision of data collectors. The interviews were conducted entirely in English. The data collection process lasted for about 10 min per participant, and the entire exercise lasted for 4 weeks (July 15–August 15, 2021). All returned questionnaires were checked for completeness.

### 2.6 Data Analysis

Data were first entered and cleaned in Microsoft Excel to ensure accuracy and consistency. The final dataset was then imported into SPSS 25.0 for statistical analysis. Descriptive statistics, such as frequencies and percentages, were used to summarize the demographic characteristics of the participants and to describe their knowledge and practice of BSE. For inferential analysis, a binary logistic regression was performed. This was used to determine the factors associated with the practice of BSE. The results were presented as both crude odds ratios and adjusted odds ratios, along with their 95% confidence intervals. The level of statistical significance for all analyses was set at a *p*‐value of less than 0.05. The findings were then presented in a clear and informative manner using tables and graphs.

### 2.7 Ethics Approval and Consent to Participate

The ethics board at Kwame Nkrumah University of Science and Technology gave their approval (ref. CHRPE/AP/346/20). Administrative approval was also granted by the Gushegu Nursing and Midwifery College. Informed consent was sought from the study participants after an in‐depth explanation of the study.

## 3 Results

### 3.1 Demographic Characteristics

The study's participants were predominantly young, with 62.8% under 25 years old and only 5.9% aged 30 or older. A significant majority, 74.4%, had never been married. The religious breakdown was fairly even, with 53.3% identifying themselves as Christians and 46.6% as Muslims. Most participants (59.8%) were registered nurse assistant clinicians. The vast majority (94.2%) had no family history of breast cancer. A notable finding was that 87.7% of participants did not perceive BSE as beneficial (Table 1).

**TABLE 1:** Socio‐demographic characteristics of participants.

Variables	Frequency (*n*)	Percentage (%)
Age		
< 25 years	86	62.8
25–30 years	43	31.4
30+ years	6	5.8
Marital status		
Never married	102	74.4
Married	35	25.6
Religion		
Christian	73	53.3
Muslim	64	46.7
Program of study		
Registered midwifery	42	30.7
Registered nurse assistant clinical	82	59.8
PNM midwifery	13	9.5
Employment status		
Unemployed	130	94.9
Employed (civil servant)	7	5.1
Physical exercise		
No	11	8.0
Yes	126	92.0
Family history of breast cancer		
Yes	8	5.8
No	129	94.2
Importance of breast self‐examination		
Not beneficial	136	87.7
Beneficial	19	12.3

### 3.2 Breast Cancer Awareness

**FIGURE 1:**
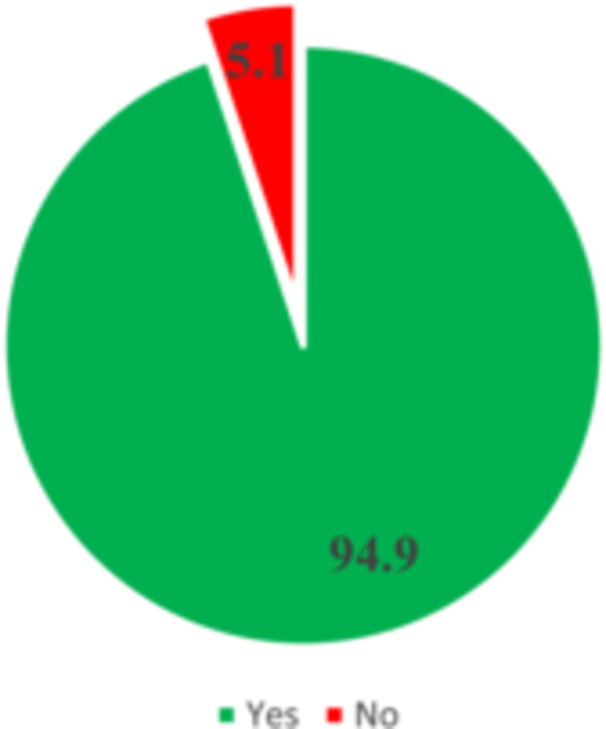
Awareness of breast cancer.

**FIGURE 2:**
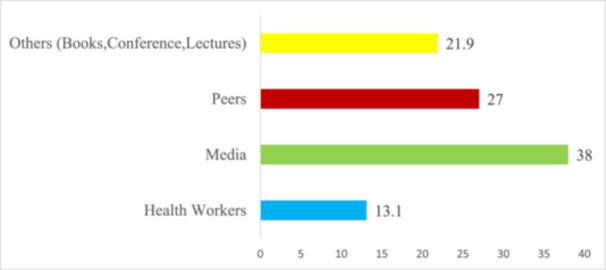
Sources of awareness of breast cancer.

Figure 1 indicates a high level of breast cancer awareness among the participants. A substantial majority, 94.9%, reported being aware of breast cancer, with only 5.1% indicating a lack of awareness. Figure 2 highlights the various channels through which participants gained information on breast cancer. The media emerged as the primary source, accounting for 38.0% of the reported information dissemination channels. Peers also played a significant role, with 27.0% of participants learning about breast cancer from them. Health workers were a source of information for 13.1% of the respondents, while a notable 21.9% acquired their information from “other sources,” including books, conferences/workshops, and academic lectures.

### 3.3 Awareness of Self‐Breast Examination

**FIGURE 3:**
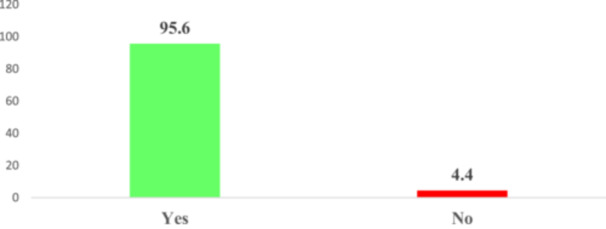
Awareness of breast self‐examinations.

**FIGURE 4:**
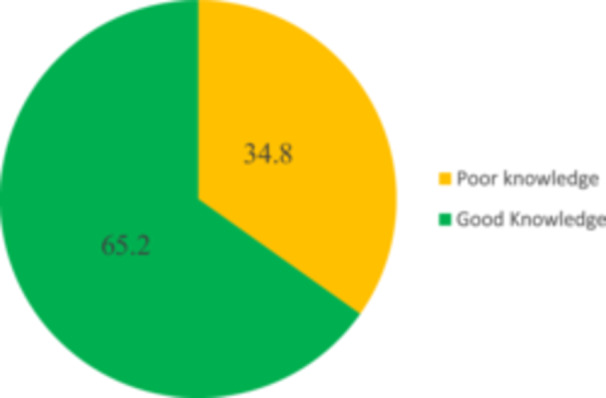
Knowledge of breast self‐examination.

**FIGURE 5:**
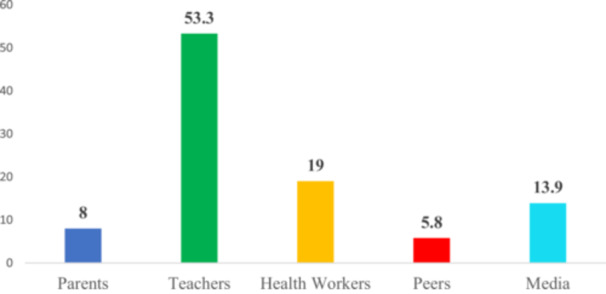
Sources of knowledge of breast self‐examination.

As illustrated in Figure 3, awareness of BSE is remarkably high among participants. The findings reveal that a significant majority, 95.6%, were aware of BSE, with only a small proportion of 4.4% reporting no awareness. Figure 4 details the level of knowledge regarding BSE among the participants. The results indicate that 65.2% of individuals demonstrated good knowledge of BSE, while a substantial 34.8% possessed poor knowledge. Figure 5 illustrates the sources of information regarding the knowledge of BSE among participants. It was found that the majority of the participants (53.3%) got their information about BSE from their teachers. About 19% of them got their information about BSE from health workers, 13.9% of the participants learned about BSE from the media, and 8.0% of them got their information about BSE from their parents. About 5.8% of them acquired their information about BSE from their peers.

### 3.4 Prevalence of Self‐Breast Examination

**FIGURE 6:**
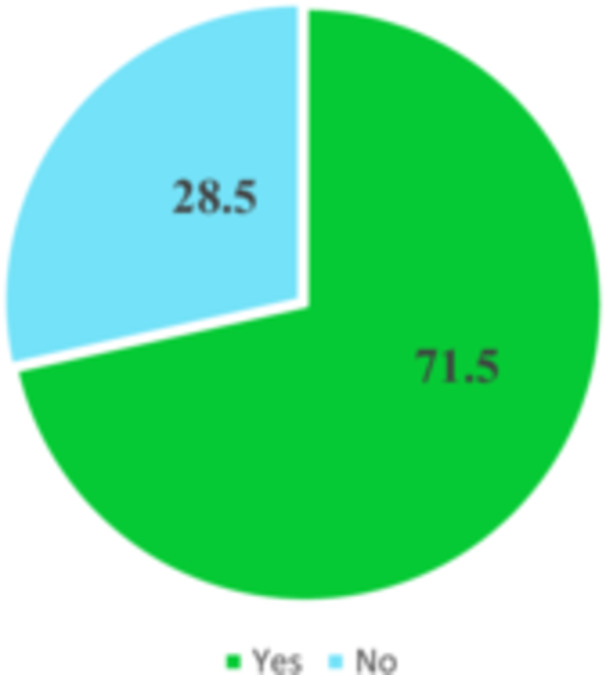
Practice of breast self‐examination.

As depicted in Figure 6, the study found that the prevalence of BSE among the participants stands at 71.2%. This shows that a significant majority of individuals in the study actively engage in BSE.

### 3.5 Factors Associated With the Practice of Self‐Breast Examination

Table 2 presents the factors associated with the practice of BSE among the participants. The results show that participants who are registered nurse assistant clinical were 0.08 times [95% CI = 0.015–0.443] less likely than those who are registered midwives to practice BSE. It was found that those participants who engaged in any form of physical exercise were 16.7 times [95% CI = 2.43–114.9] more likely to practice BSE than those participants who did not engage in any form of physical exercise. The results indicated that participants who had a family history of breast cancer had higher odds [AOR = 6.54, 95% CI 0.42–101.1] of practising BSE as compared to those who did not have a family history of breast cancer. It was noted that those participants who are aware of breast cancer have 2.98 times higher [95% CI = 0.27–32.31] probability of practising BSE than their counterparts who are not aware of breast cancer. Similarly, participants who were aware of BSE were 2.88 times [95% CI = 0.17–48.2] more likely to practise BSE than their counterparts who were not aware of BSE. Source of knowledge of BSE was found to be significantly associated with the practice of BSE among the participants, in this regard, it was noted that participants who got their information on BSE from their teachers, health worker and peers were 22.31 times [95% CI = 3.10–150.9], 28.4 times [95% CI = 3.08–262.8], 36.48 times [95% CI = 2.48–534.8] more likely to practise BSE, respectively. Finally, the results showed that those participants who perceived BSE to be beneficial had higher odds [AOR = 3.70, 95% CI = 0.67–19.96] of practising BSE than their counterparts who did not view BSE as beneficial.

**TABLE 2:** Factors associated with practice of self‐breast examination.

Variables	Practice of self‐breast examination			
	CoR [CI]	*p*‐value	AoR [CI]	*p*‐value
Age (years)				
< 25	1		1	
25–30	0.68 [0.31–1.50]	0.340		
30+	2.56 [0.28–21.91]	0.392		
Religion				
Christian	1			
Muslim	1.03 [0.49–2.17]	0.93		
Marital status				
Never married	1		1	
Married	1.20 [0.50–2.87]	0.67		
Program of study				
Registered midwifery	1		1	
Registered nurse assistant clinical	0.16** [0.51–0.48]	0.001	0.08* [0.015–0.443]	0.004
Post nurse assistant clinical/nurse assistant preventive midwifery	0.16 [0.93–3.59]	0.557	0.41 [0.040–4.21]	0.455
Employment status				
Unemployed	1		1	
Employed (civil servant)	2.48 [0.289–21.29]	0.408		

Physical exercise				
No	1			
Yes	5.14* [1.41–18.71]	0.013	16.7** [2.43–114.9]	0.004
Family history of breast cancer				
No	1		1	
Yes	2.92* [0.35–24.57]	0.032	6.54 [0.42–101.1]	0.179
Awareness of breast cancer				
No	1		1	
Yes	1.95* [0.42–9.18]	0.039	2.98* [0.27–32.31]	0.036
Awareness of breast self‐examination				
No	1		1	
Yes	5.48* [0.96–31.28]	0.050	2.88* [0.17–48.2]	0.046
Knowledge of breast self‐examination				
Poor knowledge	1		1	
Good knowledge	1.9* [0.41–2.23]	0.015	0.82 [0.26–2.61]	0.741
Source of knowledge of breast self‐examination				
Home (parents)	1		1	
School (teachers)	22.8*** [4.38–119.4]	0.000	22.31** [3.10–150.9]	0.002
Health workers	24.8** [3.83–159.7]	0.001	28.4** [3.08–262.8]	0.003
Peers	13.5* [1.47–123.74]	0.021	36.48** [2.48–534.8]	0.009
Media	2.625 [0.44–15.78]	0.292	4.48 [0.51–39.37]	0.175
Importance of breast self‐examination				
Not beneficial	1		1	
Beneficial	2.34* [0.64–8.54]	0.019	3.70* [0.67–19.96]	0.013

Abbreviations: AoR, adjusted odds ratios; CI, confidence interval; CoR, crude odds ratio.

* *p* < 0.05

** *p* < 0.01

*** *p* < 0.001; 1 = reference category.

## 4 Discussion

This study provides valuable insights into the awareness, knowledge, and practice of BSE among nursing students and the socio‐demographic factors associated with BSE in Gushegu, a rural area in northern Ghana. The remarkably high awareness of breast cancer and BSE among the participants is a positive indication, suggesting that general health education efforts have been effective in disseminating basic information on breast cancer prevention. Similarly, the finding that 65.2% of participants possessed good knowledge of BSE is encouraging. Similar high awareness/knowledge was reported among nursing and teaching, and midwifery tertiary students in Ghana, including Tamale metropolis [19, 25]. This is commendable and aligns with expectations for nursing students. As future healthcare professionals, nursing students are ideally positioned to understand health‐seeking behaviors and prevention strategies. These findings align with the broader understanding that awareness campaigns can significantly improve recognition of health issues [1, 33]. A study that is also in tandem with the current finding was conducted at the Technology Senior High School and the Kwame Nkrumah University of Science and Technology, which revealed that 90.9% of respondents were aware of BSE, and a high level of knowledge on BSE was found in 54.5% of the students [34]. However, previous literature has revealed contradictory findings [35, 36].

Despite this high awareness and knowledge of BSE, the practice of BSE was relatively moderate at 71.2%, which is comparable to findings from other studies in Africa [25, 37]. This discrepancy suggests that awareness alone may not necessarily translate into regular practice, emphasizing the importance of factors influencing behavioral change. Nonetheless, the observed prevalence of 71.2% for BSE practice is a positive finding, indicating that a significant proportion of these nursing students are engaging in this crucial self‐screening method. This contrasts with previous studies conducted among students in Ghana, which, despite strong awareness, often reported low practice rates [19, 25]. The BSE prevalence was also higher than the 52.7% [38, 39] and 37.1% in previous studies [32, 40, 41].

A study conducted in Ethiopia showed that the practice of BSE among female health professionals was low [38]. The difference could be due to variations in the knowledge and expertise of students in BSE, as well as differences in location. However, it's important to consider if this practice is regular and performed correctly, as some studies suggest a gap between reported practice and consistent, accurate technique [42, 43].

The finding that registered nurse assistant clinicians were less likely to practice BSE compared to registered midwifery participants is intriguing and warrants further investigation. This disparity could stem from differences in curriculum depth, practical exposure, or perceived professional roles regarding breast health education and self‐care. Some earlier studies did not unravel these determinants [32, 36].

Conversely, a strong synergy was observed between physical exercise and BSE practice. Consistent with prior research, this suggests that a holistic commitment to a healthy lifestyle often serves as a gateway to specific self‐care behaviors and proactive health screenings [20, 44].

Finally, a family history of breast cancer significantly increased the odds of practicing BSE. This underscores the role of heightened personal risk perception as a primary motivator for self‐examination. This finding aligns with similar evidence across the African continent, reinforcing the idea that individuals with familial exposure are more likely to adopt “cues to action” regarding their own breast health [36, 45].

Furthermore, awareness of breast cancer and awareness of BSE itself were positively linked to practice, reinforcing the foundational importance of knowledge in driving preventive actions [32, 36]. A recent study carried out among midwifery students in Nigeria found that 61% of them practiced BSE, recognizing its importance for early detection, but barriers included poor knowledge, lack of time, and privacy; exercise and family history of cancer significantly reduced the likelihood of practicing BSE and the study recommends prioritizing BSE education among student midwives to enhance early breast cancer detection [46]. Studies in Northern Ghana have also found that there is a strong positive correlation between knowledge of BSE and its practice among women [21]. Nonetheless, in the Greater Accra Region of Ghana, a recent study found that while most female nurses had adequate breast cancer knowledge, only 67% regularly practiced BSE [47].

Crucially, the source of BSE knowledge played a profound role. Participants who gained their information from teachers, health workers, and peers were significantly more inclined to practice BSE. This highlights the immense impact of trusted, direct, and relatable sources of information in translating knowledge into action. Finally, the perception of BSE as beneficial was a strong predictor of practice, underscoring the importance of reinforcing the efficacy and value of BSE in educational messages. Recent studies have consistently identified similar predictors, such as the source of BSE knowledge—particularly from teachers, health workers, and peers—and the perception of BSE as beneficial, as strong factors influencing practice [12, 19, 24, 25, 32, 37, 45]. This highlights the importance of reliable education and positive perceptions in encouraging regular BSE for early breast cancer detection.

## 5 Conclusion

In conclusion, this study among nursing and midwifery students in Gushegu, northern Ghana, revealed a high level of awareness and knowledge regarding breast cancer and BSE. The observed BSE practice prevalence of 71.2% is commendable, particularly for a rural setting, and may reflect the impact of professional training among these future healthcare workers. Key factors associated with BSE practice include engagement in physical exercise, a family history of breast cancer, and awareness of both breast cancer and BSE. Crucially, the source of BSE knowledge, particularly from teachers, health workers, and peers, strongly predicted practice. Despite high awareness, a significant proportion of participants initially did not perceive BSE as beneficial, highlighting a critical attitudinal barrier. Therefore, future interventions should not only maintain high awareness and knowledge levels but also actively reinforce the perceived benefits of BSE through trusted educational channels and address any specific professional or attitudinal barriers to ensure consistent and effective breast cancer screening practices.

### 5.1 Study Limitations

While the cross‐sectional design of this study precludes the establishment of definitive causal relationships, the findings offer a vital snapshot of current trends. We acknowledge that data regarding awareness, knowledge, and BSE practices rely on self‐reports, which may introduce recall or social desirability bias. Furthermore, although the relatively modest sample size may constrain the broad generalizability of the findings, the participant cohort is sufficiently representative of the target demographic to maintain the internal validity of our conclusions. Ultimately, the evidence generated is consistent with existing literature and provides a substantive foundation to inform evidence‐based policy decisions.

## Author Contributions

**Robert Kokou Dowou:** writing – review and editing, methodology, validation, formal analysis, software.

## Funding

The authors have nothing to report.

## Disclosure

The lead author Gifty Apiung Aninanya affirms that this manuscript is an honest, accurate, and transparent account of the study being reported; that no important aspects of the study have been omitted; and that any discrepancies from the study as planned (and, if relevant, registered) have been explained.

## Consent

The authors have nothing to report.

## Conflicts of Interest

The authors declare no conflicts of interest.

## Data Availability

The data that support the findings of this study are available from the corresponding author upon reasonable request.
